# Potential of *Artemisia annua* Bioactives as Antiviral Agents Against SARS-CoV-2 and Other Health Complications

**DOI:** 10.3390/ph18121904

**Published:** 2025-12-17

**Authors:** Nehad A. Shaer, Amal A. Mohamed, Ewald Schnug

**Affiliations:** 1 Department of Biology, Jamoum University College, Umm Al-Qura University, Makkah 21955, Saudi Arabia; nashaer@uqu.edu.sa; 2 Department of Chemistry, Al-Leith University College, Umm Al-Qura University, Makkah 21955, Saudi Arabia; 3 Department of Plant Physiology and Biochemistry, Faculty of Life Sciences, Technical University of Braunschweig, D-38106 Braunschweig, Germany; e.schnug@tu-bs.de

**Keywords:** *Artemisia annua*, antiviral compounds, COVID-19, medicinal plants

## Abstract

This review highlights *Artemisia annua*, a medicinal plant which grows in the Kingdom of Saudi Arabia, known for its abundant therapeutic properties. *A. annua* serves as a rich source of various bioactive compounds, including sesquiterpenoid lactones, flavonoids, phenolic acids, and coumarins. Among these, artemisinin and its derivatives are most extensively studied due to their potent antimalarial properties. Extracts and isolates of *A. annua* have demonstrated a range of therapeutic effects, such as antioxidant, anticancer, anti-inflammatory, antimicrobial, antimalarial, and antiviral properties. Given its significant antiviral activity, *A. annua* could be investigated for the development of new nutraceutical bioactive compounds to combat SARS-CoV-2. Artificial Intelligence (AI) can assist in drug discovery by optimizing the selection of more effective and safer natural bioactives, including artemisinin. It can also predict potential clinical outcomes through in silico modeling of protein–ligand interactions. In silico studies have reported that artemisinin and its derivatives possess a strong ability to bind with the Lys353 and Lys31 hotspots of the SARS-CoV-2 spike protein, demonstrating their effective antiviral effects against COVID-19. This integrated approach may accelerate the identification of effective and safer natural antiviral agents against COVID-19.

## Simple Summary

Extracts and bioactive compounds from *Artemisia annua* have demonstrated activity against SARS-CoV-2 in laboratory studies and cell-based assays. Compounds like artemisinin showed potential to inhibit key viral proteins like the main protease (Mpro), interfering with virus replication. Rutin and quercetin derivatives, present in Artemisia extracts, have antiviral effects against SARS-CoV-2 and other viruses. Beyond direct antiviral activity, Artemisia extracts exhibit immunomodulatory and anti-inflammatory effects, potentially reducing COVID-19-associated inflammation and oxidative stress.

## 1. Introduction

In traditional medicine, medicinal plants are a valuable source of drugs that are useful in treating a wide range of illnesses. Bioactive compounds are natural substances that can have beneficial effects on living organisms. In plants, these compounds act as a defense mechanism, helping to protect against pests, diseases, and harsh environmental conditions. For humans, bioactive compounds offer significant health benefits, including antioxidants, antimicrobial, anti-inflammatory, and anticancer properties [1]. In Saudi Arabia, people have relied on medicinal plants alongside modern medicine for centuries to treat various health issues. For instance, *Nigella sativa*, commonly known as black seed, is traditionally used to boost the immune system and address respiratory problems [2]. *Ziziphus spina*-christi is well-known for its effectiveness in managing diabetes and digestive disorders [2]. Additionally, *Aloe vera* is frequently applied to relieve skin issues and promote healing. This combination of traditional herbal remedies and conventional treatments reflects the strong cultural trust in natural medicine throughout Saudi Arabia and highlights its significant role in healthcare today [3]. In real terms, traditional Islamic and Arab medicine dates back thousands of years and is a well-known form of treatment in many of these nations. In order to remedy, diagnose, and prevent illnesses as well as preserve overall health, this traditional medicine refers to healing practices and philosophy that integrate herbal medications, spiritual therapies, and dietary practices [2,3]. Acute respiratory distress syndrome is the outcome of immunological dysregulation and cytokine storm brought on by the release of inflammatory cytokines in COVID-19 patients. COVID-19 is caused by severe acute respiratory syndrome coronavirus 2 (SARS-CoV-2) and is an ongoing public health emergency [4].

Adopting natural products (NPs) may enhance immunity and offer protection against negative effects safely, often resulting in fewer side effects compared to synthetic medications. The research that is currently available supports the use of NPs against SARS-CoV-2 because of their immune-supportive, antioxidant, antiviral, anti-inflammatory, and preventative properties. Natural products regulate immune function by controlling adaptive immunity in a multidirectional fashion, as stated by Boozari and Hosseinzadeh [4].

## 2. An Overview of Coronavirus Pathogenesis and Its Life Cycle

**Figure 1:**
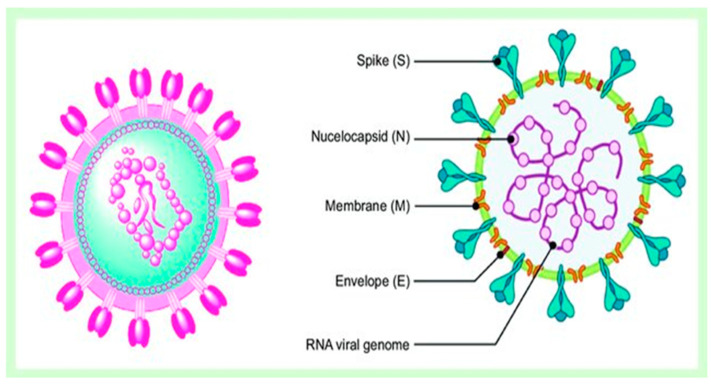
The diagram represents the SARS-CoV-2 structure showing the outer envelope, featuring its characteristic spike glycoprotein S, the virus capsid that protects the nucleic acid inside, and the M and E proteins. This figure was uploaded by Murtaza Tambuwala (https://www.researchgate.net/profile/Murtaza-Tambuwala (accessed on 2 December 2025)).

The broad family of coronaviruses (CoV) is composed of several different genera, including α, β, γ, and δ [5]. These viruses are single-positive-strand RNA viruses that exhibit varying levels of pathogenicity and immunogenicity. They can infect both humans and animals, leading to illnesses affecting various systems such as the gastrointestinal tract, liver, brain, and respiratory system [6]. The recent outbreak of the novel strain SARS-CoV-2 (formerly 2019-nCoV) has garnered significant global attention. This pathogen, which is the source of COVID-19, poses a serious health risk, particularly for the elderly [7]. SARS-CoV-2 (Figure 1) is highly pathogenic and has already infected over 178 million individuals, resulting in approximately 4 million deaths worldwide as of June 2021 [8]. The pathological characteristics of COVID-19 can vary in terms of their distribution and severity. While most patients experience respiratory tract and pulmonary symptoms, those with severe disease may also develop life-threatening conditions such as widespread thrombosis in small and large blood vessels, microvascular injury, cardiac conduction abnormalities, neurologic deficits, diarrheal symptoms, and gastrointestinal bleeding [9]. The exact mechanisms underlying these disease manifestations are unclear. However, existing data suggest that severe COVID-19 is a result of a combination of immunological dysregulation, fatigue of crucial immune cell subsets, complement activation, and interactions between the innate immune system and the coagulation cascade, which stimulate inflammatory thromboses [10].

SARS-CoV-2 is transmitted between individuals through aerosol droplets expelled by infected individuals during sneezing, coughing, or other activities that release sputum. To enter the alveolar epithelial cells of the lungs, SARS-CoV-2 utilizes the viral spike protein, which interacts with the angiotensin-converting enzyme-2 (ACE-2) receptor. Subsequent to binding, the virus gains entry into the cell via endocytosis. Once inside the cell, the viral particle exploits cellular machinery to undergo multiplication and replication [9,10]. Further investigation and exploration into the virus and the immune system is imperative in order to enhance our comprehension of the precise mechanism by SARS-CoV-2 infection develops, and ultimately result in advancements in accurate identification, management, and the development of a potent vaccine SARS-CoV-2 to prevent COVID-19.

## 3. Plant’s Derived Compounds with Potential to Combat the Coronavirus

Since they are safe, effective, have few toxic effects, and have potent therapeutic prospects, medicinal plants have been utilized effectively in traditional medical practices for centuries. Plant-derived phytochemicals, including phenolic acids, flavonoids, saponins, lignans, terpenoids, and carotenoids, as well as essential oils, are exciting categories that have substantial applications in the dietary supplement, pharmaceuticals, and agricultural industries [11,12,13,14,15,16,17,18]. Nonetheless, viral infections undoubtedly represent the greatest risk of a pandemic in today’s world among the numerous infectious disease outbreaks that humanity has had to deal with. This highlights the value of medicinal plants in treating SARS-CoV-2 infections causing COVID-19. Secondary metabolites, including lignans, flavonoids, alkaloids, coumarins, phenols, quinones, saponins, and terpenes, are found in a wide variety of living organisms and exhibit antiviral properties by blocking distinct viral replication stages [16]. Recent studies have reported on their various antiviral mechanisms, including reverse transcription, protease and integrase inhibition, virus adsorption, and virus–cell fusion [19]. Essential oils have a potent ability to disrupt the viral envelope or cover up viral components that are required for the virus to bind to or enter host cells [20]. Moreover, plant metabolites can inhibit cellular signaling pathways, stop the fusion of the coronavirus S-protein, and block enzyme activity involved in the replication cycle of coronaviruses, such as papain-like protease [21].

The majority of the Arabian Peninsula is covered by the Kingdom of Saudi Arabia, which extends 2,250,000 km^2^. Most of the country is arid land areas with a high species diversity, especially medicinal plants [17,18].

Researchers have been looking for new compounds from medicinal plants with the potential to combat COVID-19. In light of this, the purpose of this review is to discuss possible metabolites from *Artemisia annua*, a KSA medicinal plant that may be compatible with the alternate strategy against SARS-CoV-2 causing COVID-19 due to their antiviral properties.

## 4. Artemisia Annua

### 4.1. Morphology and Distribution

**Figure 2:**
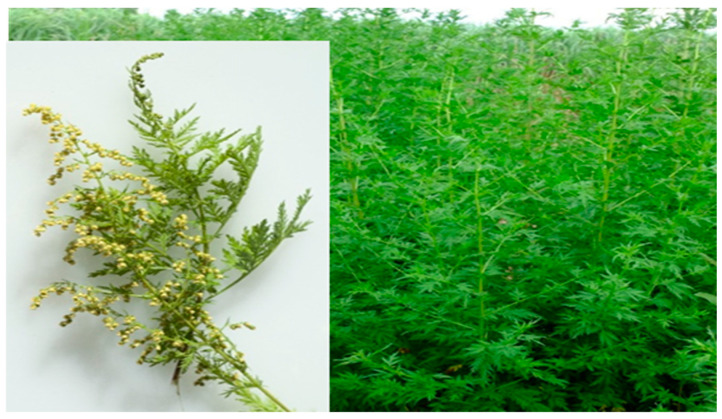
*Artemisia annua*, stems, leaves, and flowers. (https://www.laboiteagraines.com/boutique/fleurs/graines-fleurs-annuelles/artemisia-annua-bio/ (accessed on 2 December 2025); https://www.heistek-mht.nl/onze-praktijk/diverse-%28medische%29-artikelen/artemisia-annua/ (accessed on 2 December 2025)).

The *Artemisia* L. genus includes annual or perennial flowering herbs and small shrubs from the Asteraceae family. These plants are known locally as wormwood, sagewort, and sagebrush, and there are approximately 500 species distributed across Asia, Africa, Australia, Central and South America, and Europe [22]. These plants demonstrated noteworthy economic and ecological benefits due to their valuable ethnobotanical uses, such as medicinal herbs, food sources in many areas of the world, and livestock feed [23]. *Artemisia annua* (Figure 2) is one of the most important plants of the genus Artemisia, which is a fragrant flowering herb with an erect stem. The leaves, which measure 3–5 cm in length, are entirely divided into two or three leaflets. The blooms are small, greenish-yellow, and approximately 2–2.5 mm in diameter, arranged in loose panicles. It is widely used as a culinary spice, herbal tea, and medicinal plant around the world [24]. In addition to A. annua, four additional species of Artemisia grow in Saudi Arabia, namely *A. monosperma*, *A. sieberi*, *A. Judaica*, and *A. scoparia* [25].

### 4.2. Phytochemical Constituents

**Figure 3:**
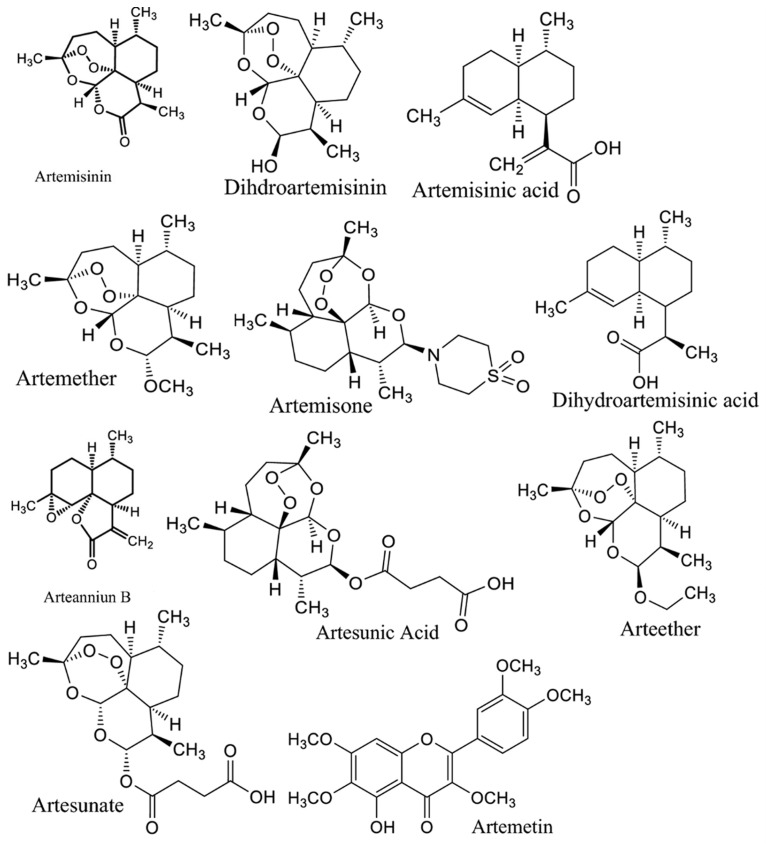
Major sesquiterpene lactones in *A. annua*.

**Figure 4:**
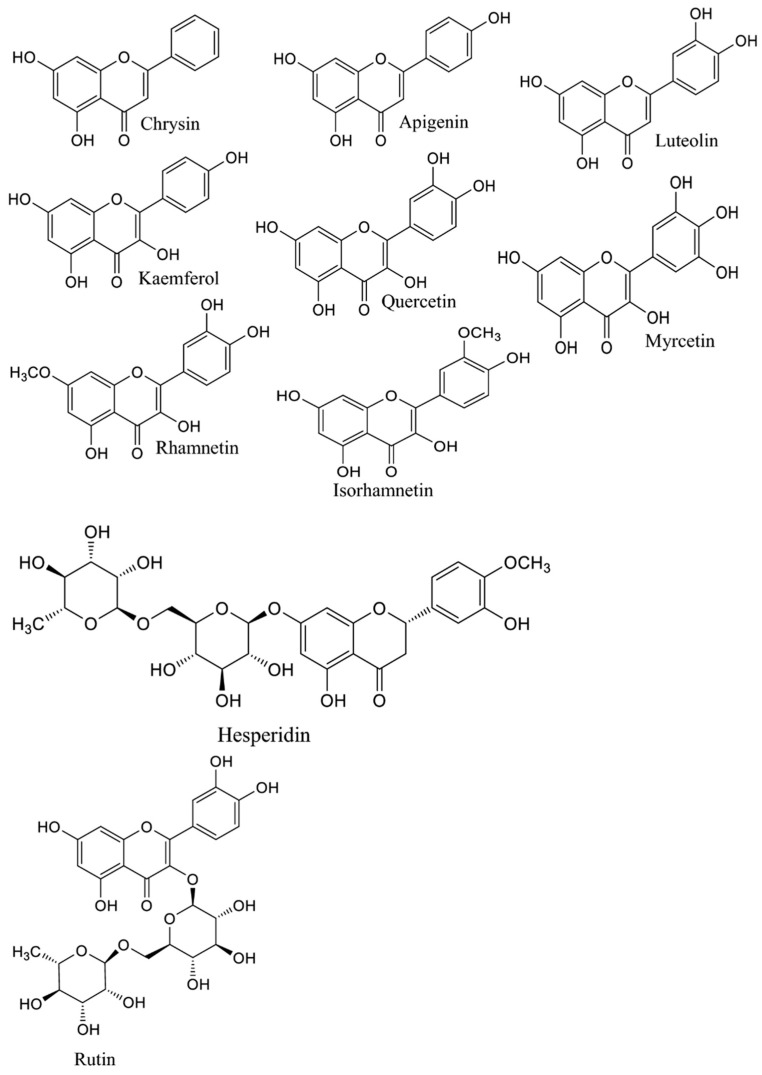
Major flavonoids in *A. annua*.

**Figure 5:**
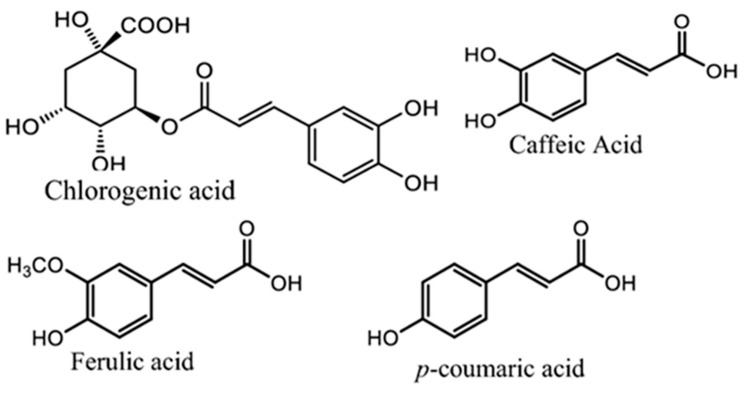
Major phenolic acids in *A. annua*.

**Figure 6:**
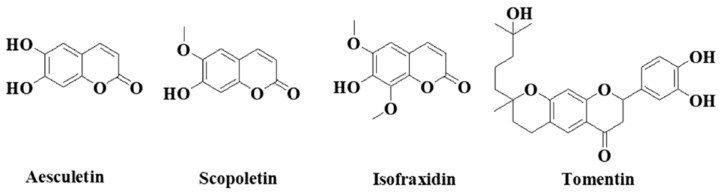
Major coumarins in *A. annua*.

*A. annua* contains abundant bioactive molecules. Artemisinin and its derivatives, the antimalarial bioactive lactone sesquiterpenes isolated from *A. annua*, are the most extensively studied. Moreover, many bioactive ingredients of various categories, which are presented in Table 1, including sesquiterpenoid lactones (Figure 3), flavonoids (Figure 4), phenolic acids (Figure 5), and coumarins (Figure 6), have been identified in the different extracts of *A. annua* [25,26,27]. In addition, many bioactive terpenes have been identified in the essential oil of *A. annua* [28,29]. Moreover, Brisibe et al. [30] demonstrated that *A. annua* is a rich source of nutritious phytochemicals, such as vitamins, minerals, amino acids, and other essentials for good health. Various sesquiterpene lactones have been identified in the aerial parts of *A. annua*; the major ones are artemisinin, artemisinic acid, and arteannuin B [31]. Quercetin, luteolin, apigenin, rutin, kaempferol, isorhamnetin, and casticin represent the major flavonoids in the aqueous and alcoholic extracts of *A. annua*, in addition to the phenolic acids of quinic acid, chlorogenic acid, and caffeic acid [31]. The alcoholic extracts of *A. annua* contain various bioactive coumarins, the main ones being scopoletin and scopoline [31]. 1, 8-Cineole, camphene, borneol, α-pinene, camphor, limonene, α-terpinene, carvone, and myrtenol represent the major terpenes found in the *A. annua* essential oils [28,29].

### 4.3. Therapeutic Potential of A. annua

**Figure 7:**
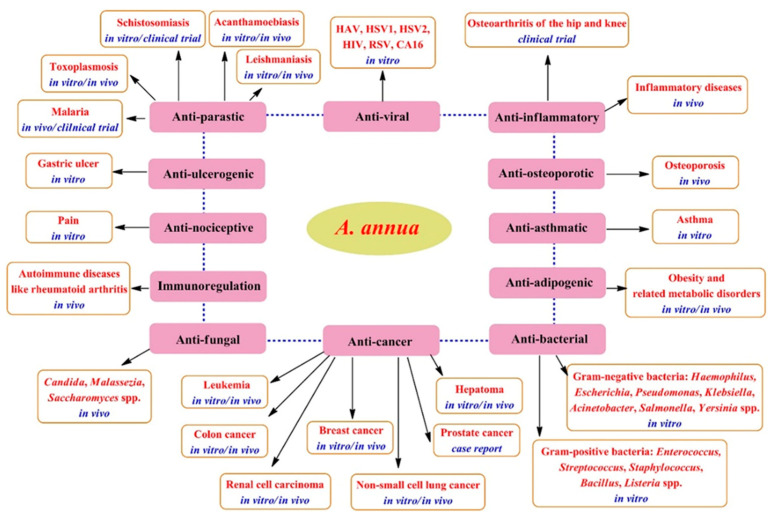
Therapeutic potentials of *A. annua* [31].

Artemisia species have been greatly utilized in traditional medicine to effectively remedy various illnesses, for example, malaria, typhoid fever, intestinal disorders, pneumonia, morning sickness, kidney problems, and epilepsy [3]. *A. annua* is significantly utilized in traditional Asian medicine to remedy wounds, jaundice, hemorrhoids, and bacterial dysentery. Moreover, it serves as a potent antipyretic drug for tuberculosis and malaria, as well as other bacterial, viral, and autoimmune illnesses [27]. Various pharmacological investigations have shown the medicinal benefits of *A. annua* extracts and essential oils (Figure 7), including their outstanding ability to promote the immune system and exert strong antioxidants, anti-inflammatory, anti-cancer, antimicrobial, antiviral, and antiparasitic effects, as extensively reviewed by Feng et al. [31], Septembre-Malaterre et al. [36], and Ekiert et al. [27].

### 4.4. Antioxidant Potentials of A. annua

Numerous investigations have revealed the potent antioxidant potential of *A. annua* essential oils and extracts, which might be caused by its abundance of bioactive ingredients of flavonoids, coumarins, and terpenes [37]. The crude ethanolic extract of A. annua and its fractions showed potent antioxidant potential using in vitro tests of iron (II) chelating, ferric reducing antioxidant power (FRAP), cupric reducing antioxidant capacity (CUPRAC), DPPH, and ABTS; the superior effects were for the crude extract compared to its fractions [37]. *A. annua* extracts and essential oils from different countries showed antioxidant effects in in vitro assays, including DPPH, ABTS, Oxygen Radical Absorbance Capacity (ORAC), and metal chelation [38,39].

### 4.5. Anti-Inflammatory Potentials of A. annua

*A. annua* extracts and their major bioactive artemisinin demonstrated strong anti-inflammatory potentials in both in vitro and in vivo models. *A. annua* tea solution lowered inflammation in Caco-2 cells by suppressing IL-6 and IL-8 generation, probably thanks to the presence of rosmarinic acid, a major phenolic molecule identified in the extract [40]. The aqueous, methanolic, ethanolic, and acetone extracts of *A. annua* effectively reduced inflammation in LPS-induced RAW 264.7 macrophages at a concentration of 100 µg/mL [41]. The acetone extract with the highest artemisinin concentration had the maximum inhibitory impact on the production of pro-inflammatory cytokines (IL-1, IL-6, and IL-10), prostaglandin E2 (PGE2), and nitric oxide (NO) [41]. In LPS-induced RAW 264.7 macrophages, the ethanol extract of *A. annua* and its isolated compounds: scopoletin, artemisinin, chrysosplenetin, 3-O-β-D-glucopyranoside of sitosterol, and eupatin, effectively inhibited inflammation by preventing the release of NO [42]. In croton oil-induced edema and LPS-induced inflammation in mice, casticin and chrysosplenol D, two major flavonoids isolated from *A. annua*, revealed dose-dependent anti-inflammatory effects. They successfully suppressed the generation of inflammatory mediators by regulating NF-κB and c-JUN, verifying their effective role in inhibiting inflammation [43]. The bioactive sesquiterpenes lactone artemisinin and its derivatives, which are extensively isolated from *A. annua*, have established strong anti-inflammatory potentials in numerous inflammatory disease models, including allergic inflammation, septic inflammation, and autoimmune diseases. These effects arise through the downregulation of various pathways: the PI3K/Akt signalling cascade, the mitogen-activated protein kinase (MAPK) pathway, the expression of Toll-like receptors 4 (TLR4) and 9 (TLR9), and NF-κB activation [44].

### 4.6. Immunomodulatory Potentials of A. annua

*A. annua* has a long history of utilization in ancient China for efficiently treating various autoimmune diseases, including rheumatoid arthritis, revealing its effective immunoregulatory properties [43]. *A. annua* ethanol extract efficiently suppressed immunological responses by inducing lymphocyte proliferation in mouse splenocytes initiated by lipopolysaccharide (LPS) and concanavalin A. Additionally, when administered intraperitoneally, the *A. annua* extract significantly reduced blood levels of IgG, IgG1, and IgG2b antibodies in mice that had been immunized with ovalbumen. It also prevented splenic enlargement [45]. The aqueous extract of *A. annua* showed substantial immunomodulatory ability by actively modulating essential components of the innate immune system, particularly Toll-like receptors TLR2 and TLR4 in the lungs and brains of mice infected with Acanthamoeba [46]. Numerous pharmacological studies demonstrated the substantial immunomodulatory potentials of artemisinin and its derivatives, dihydroartemisinin, artesunate, and artemether, extensively isolated from *A. annua* [44,47]. These compounds effectively influence the responses of various innate and adaptive immune cells. They significantly inhibit cytokine release and related signaling pathways, reduce neutrophils, suppress macrophage functionality, inhibit lymphocyte production and conservation, and influence various signaling paths, such as TLRs, Akt, PLCγ, MAPK, PKC, STATs, Wnt, Nrf2/ARE, and NF-κB [36,47].

### 4.7. Antimicrobial Potential of A. annua

Many research studies have demonstrated the antimicrobial abilities of *A. annua* essential oils and extracts against a variety of bacteria and fungi, including Gram-positive species of Streptococcus, Enterococcus, Enterococcus, Listeria, Staphylococcus, and Bacillus; Gram-negative species of Escherichia, Klebsiella, Pseudomonas, Acinetobacter, Haemophilus, Salmonella, and Yersinia; and fungi species of Candida, Saccharomyces, and Malassezia [26,27,28]. The essential oil of *A. annua* demonstrated remarkable antimicrobial and antibiofilm properties, with minimum inhibitory concentrations (MICs) ranging from 0.51 to 16.33 mg/mL. It excellently combated a range of clinical microbes, including the Gram-negative bacteria *P. aeruginosa*, *E. coli*, *K. pneumoniae*, and *A. baumannii*; Gram-positive bacteria *S. aureus*, *B. subtilis*, and *E. faecalis*; and yeasts *C. famata*, *C. utilis*, and *C. albicans*. Camphor, α-pinene, germacrene D, 1,8-cineole, trans-β-caryophyllene, and artemisia ketone represented the major constituents of *A. annua* essential oil and might be associated with its antibacterial properties [28]. The essential oil of *A. annua* demonstrated remarkable abilities to combat a variety of Malassezia fungi, including *M. globosa, M. furfur, M. pachydermatis*, *M. sloffiae, and M. sympodialis*, as well as Candida fungi, including *C. krusei*, *C. dubliniensis*, *C. parapsilosis*, *C. albicans*, *C. glabrata*, *C. tropicalis*, and *C. norvegensis*. The major components of *A. annua* essential oil, camphor, 1,8-cineole, and artemisia ketone are responsible for its antifungal properties [48].

### 4.8. Anti-Malarial Potential of A. annua

Various reports have highlighted the anti-malarial properties of *A. annua*, leading to different herbal medications made from *A. annua* that are proposed for the inhibition and treatment of malaria [49,50,51]. Various clinical trials have verified that *A. annua* tea preparations efficiently alleviate malaria symptoms, with the cure rate directly associated with the dosage administered [49]. Artemisinin concentrations as low as 9 ng/mL have been established to suppress the growth and development of *P. falciparum*. Pharmacokinetic findings indicate that plasma artemisinin levels after drinking *A. annua* tea reach 9 ng/mL and continue at that level for at least four hours. This suggests that *A. annua* tea may contain abundant artemisinin to have effective therapeutic antimalarial properties [50]. Because of the high efficiency of artemisinin against *P. falciparum*, the World Health Organization (WHO) advises using artemisinin-based combination treatment (ACT) for treating uncomplicated malaria [51].

### 4.9. Antiviral Potentials of A. annua

Massive studies have confirmed the strong antiviral effectiveness of *A. annua* extracts and bioactive artemisinin derivatives against a range of DNA and RNA viruses [26,36]. Various extracts of *A. annua* have been evaluated against herpes simplex viruses (HSV), which are responsible for a range of human ailments, from severe encephalitis to herpes labialis. In HeLa cells, the methanol extract of *A. annua* displayed promising antiviral efficacy against HSV-1, superior to the effectiveness of acyclovir even at a low dose of 3.125 µg/mL [52]. Even though extracts such as petroleum ether, ethyl acetate, and n-butanol did not affect HSV-2, the aqueous extract of *A. annua* showed potent antiviral efficiency against HSV-2 in Vero cells, similar to that of acyclovir [53]. The main compounds identified in the aqueous extract were polyphenols and condensed tannins, which are supposed to be responsible for its anti-HSV properties [53]. A study by Lubbe et al. [54] established that an infusion of *A. annua* tea successfully inhibited human immunodeficiency virus (HIV) in vitro with an IC_50_ value of 2.0 µg/mL. Furthermore, *A. annua* extract and essential oil have established potent antiviral efficiency against Hepatitis A virus (HAV), coxsackievirus type A16 (CA16), and respiratory syncytial virus (RSV) [26,55]. Numerous studies have displayed the significant antiviral efficiency of artemisinin and its derivatives, the major bioactive constituents in *A. annua*, against various viruses, including HSV-1, HSV-2, human herpesvirus 6, bovine viral diarrhea, and hepatitis B virus [56].

### 4.10. A. annua Potential in Combating SARS-CoV-2

**Figure 8:**
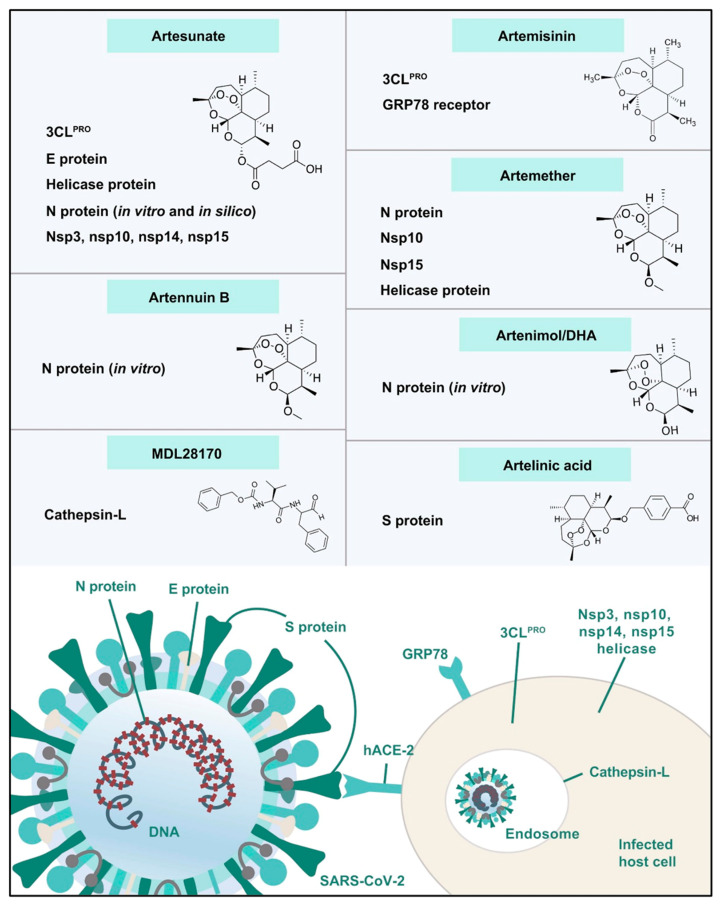
Artemisinin-based compounds as potential COVID-19/host protein inhibitors [66].

Many studies have found that *Artemisia* spp. contains enough bioactive phytochemicals, especially polyphenols and artemisinin derivatives, which effectively combat viruses [26,36]. A growing body of research emphasizes the powerful efficiency of *A. annua* extracts and their bioactive ingredients, especially artemisinin, in combating coronaviruses. This promising indication shows the potential of these natural components in treating viral challenges. Lin et al. [57] first reported the effectiveness of *A. annua* against the SARS coronavirus that emerged in 2002. *A*. *annua* showed high efficiency to combat SARS-CoV-2 through inhibiting its replication and penetration with a EC_50_ of 1053.0 µg/mL and EC_50_ of 34.5 µg/mL [58]. In a recent in silico study, Sehailia and Chemat [59] reported that artemisinin and its derivatives demonstrate superior antiviral effects against SARS-CoV-2 when compared to hydroxychloroquine (HCQ). This is due to their potent capacity to combine with the Lys353 and Lys31 hotspots of the SARS-CoV-2 spike protein. The study found that artenilic acid achieved a better Vina docking score of −7.1 kcal/mol, while hydroxychloroquine had a score of −5.5 kcal/mol. The treatment with *A. annua* extract, plus artemisinin, artemether, and artesunate, significantly reduced SARS-CoV-2 infection in vitro infection in Vero E6 cells, human lung cancer A549-hACE2 cells, and human hepatoma Huh7.5 cells. The efficiency of these treatments against COVID-19 was arranged as follows: artesunate > artemether > *A. annua* extract > artemisinin [60]. The hot aqueous extracts of five cultivars of *A. annua* exhibited effective in vitro antiviral potentials against various COVID-19 variants, including alpha, beta, gamma, delta, and kappa. The IC_50_ values ranged from 0.3 to 8.4 µM, while the IC_90_ values ranged from 1.4 to 25 µM. These results were compared to the positive control, artemisinin [61]. Extracts from Artemisia exhibited a potent ability to inhibit SARS-CoV-2 infection in vitro and Feline coronavirus (FCoV) at lower concentrations that did not affect cell efficacy and viability [62]. Abundant studies reported the potent antiviral efficiency of artemisinin and artemisinin-based compounds, including arteannuin B, artesunate, artemether, and dihydroartemisinin, against various COVID-19 variants [63,64]. Extracts of *A. annua*, artemisinin, and artemisinin-based compounds demonstrate various mechanisms that are responsible for their prospective anti-COVID-19 effects. These mechanisms include preventing the binding of COVID-19 spike proteins to host cell surfaces, thus inhibiting both the endocytosis of the virus and the activation of the NF-κB signaling pathway [59,65]. Artemisinin-based compounds demonstrated strong efficacy in binding to various COVID-19-host proteins, including the E protein, 3CLPRO, helicase protein, S protein, N protein, nonstructural protein 3 (nsp3), nsp10, nsp14, nsp15, the glucose-regulated protein 78 receptor, and cathepsin L, as illustrated in Figure 8 [66]. *A. annua* and its bioactive phyto-components have the potential to combat SARS-CoV-2 infection (COVID-19) by promoting the host’s adaptive immunity. They do this by producing CD8 and CD4 lymphocytes, which are responsible for generating antibodies that target the virus. Additionally, these components help to suppress the generation of pro-inflammatory cytokines, including TNF-α, prostaglandin E2 (PGE2), and interleukins (IL-6) and (IL-10). This process can lead to an increased CD4 count and a higher CD4/CD8 ratio [23]. Moreover, artemisinin-based compounds may also help combat COVID-19 by mitigating cytokine storms in severe COVID-19. They achieve this by inhibiting IκB kinase (IKK) and, therefore, reducing intense NF-κB signaling. Alternatively, these compounds may prevent the transcriptional action of the p50/p65 dimer that is released during NF-κB signaling [65].

### 4.11. Some Clinical Trials of Artemisia annua and Its Products

Various in vivo findings demonstrated that *A. annua* extract and its isolation, artemisinin, can decrease inflammatory reactions of cytokines like Tumor Necrosis Factor- alpha (TNF-α) and Interleukin-6 (IL-6). These cytokines are of key cytokines that have been reported in “cytokine storm” caused harsh problems in SARS-CoV-2 patients experience. Additionally, artemisinin can reduce fibrosis, a severe condition that SARS-CoV-2 patients face, and can cause organs damages [67]. Consequently, Latarissa et al. [67], concluded that *A. annua* extract and its isolation, artemisinin, can combat SARS-CoV-2 through preventing the progress of cytokine storm and organs fibrosis. Moreover, the clinical trials assessing by Li et al. [68] found that antimalarial drugs, including Artemisinin–Piperaquine (AP), for COVID-19 were designed as randomized controlled trials focusing on evaluating the efficacy and safety of single antimalarial agents without combination therapies. These trials involved COVID-19 patients confirmed by PCR testing, mostly adults aged 18 years and older, with varying disease severities from mild to moderate. The studies compared the antimalarial interventions such as Quinine Sulfate (QS), Atovaquone (AQ), and Artemisinin–Piperaquine (AP), against control groups receiving either standard of care plus placebo or other comparators [68].

The primary outcomes typically focused on viral clearance measured by time to reach undetectable levels of SARS-CoV-2, while secondary outcomes included clinical status scales, oxygen supplementation incidence and duration, mechanical ventilation need, length of hospital stay, and safety indicators such as QT interval prolongation on electrocardiograms and adverse events. Among the drugs evaluated, Artemisinin–Piperaquine significantly shortened the time to viral clearance compared to the control group (10.6 days versus 19.3 days). However, it was also associated with a notable increase in the QT interval, underscoring the need for vigilant cardiac monitoring. In contrast, trials involving Quinine Sulfate and Atovaquone did not demonstrate statistically significant improvements in both primary and secondary outcomes, although some descriptive findings suggested possible benefits.

It is important to note that the primary outcomes in the clinical study were the primary endpoints that assessed the drug’s efficacy directly. Although they were not the primary focus of the trial, secondary outcomes comprised further measurements that provided additional information about the medication’s effects and safety.

In vivo studies by Xu et al. [69] investigated the enhanced therapeutic effects of whole-plant extracts of *A. annua* compared to pure artemisinin. The researchers prepared natural artemisinin-based combination therapies (nACTs) and tested their biological activity in two murine malaria models, where nACTs showed a roughly tenfold greater antimalarial effect than artemisinin alone at equivalent doses. Pharmacokinetic studies in rats revealed that oral administered nACTs prolonged half-life, and extended mean residence time, indicating slower drug elimination and better systemic absorption. Cellular assays using Caco-2 cells demonstrated that the extracts modulated P-glycoprotein activity and reduced artemisinin efflux, primarily through components such as deoxyartemisinin, artemisinic acid, and dihydroartemisinic acid [69].

*A. annua* extracts (nACTs) demonstrated approximately ten times greater in vivo antimalarial activity than pure artemisinin at the same dose, according to the findings published by Xu et al. [69]. Since the extracts contain other bioactive components such deoxyartemisinin, artemisinic acid, and dihydroartemisinic acid, this improvement is thought to be the result of several causes acting together. These bioactive substances are known to improve the absorption and retention of artemisinin by blocking P-glycoprotein (P-gp), a protein that pumps medications out of cells. Additionally, the extracts increase artemisinin’s half-life and oral bioavailability, extending its duration in the bloodstream.

In addition, *A. annua* contains also a wide range of bioactive compounds, which include flavonoids, phenolic acids, monoterpenes, sesquiterpenes, coumarins, and essential oils which are considered to have powerful antimalarial activities [70]. Preclinical and experimental evidence reported that these compounds contribute to a broad spectrum of pharmacological effects such as antimicrobial, antiviral, anticancer, anti-inflammatory, antioxidant, antihypertensive, antidiabetic, hepatoprotective, and insect-repellent activities [70]. The mechanisms underlying these activities involve oxidative stress modulation, disruption of microbial and parasitic pathways, and effects on cellular signaling pathways (e.g., nuclear factor kappa B, cyclooxygenase and lipoxygenase enzymes, and the Janus kinase-signal transducer and activator of transcription signaling pathway, NF-κB, COX/LOX, JAK-STAT).

A randomized controlled clinical trial by involving 159 patients with active rheumatoid arthritis (RA) was conducted, dividing participants into two groups: 80 in the control group and 79 in the *A. annua* extract (EAA) group. Over 48 weeks, the control group received standard treatment with methotrexate and leflunomide, while the EAA group received the same drugs supplemented with 30 g/day of EAA. Significant improvements in pain scores, number of tender joints, and erythrocyte sedimentation rate (ESR) were observed in the EAA group as early as 12 weeks. By 24 and 48 weeks, the EAA group demonstrated statistically significantly greater overall treatment efficacy compared to controls. Additionally, corticosteroid discontinuation rates were higher, and adverse events were reduced within 12 weeks in the EAA group. These findings indicate that combining EAA with methotrexate and leflunomide enhances therapeutic outcomes in active RA patients [71].

Artemisinin derivatives from *A. annua* also help regulate immune balance by modulating the ratio of T helper 17 (Th17) cells to regulatory T (Treg) cells, which plays a central role in autoimmune arthritis. These compounds interfere with the migration and invasiveness of fibroblast-like synoviocytes (FLS) by targeting signaling pathways such as phosphoinositide 3-kinase/protein kinase B/mammalian target of rapamycin (PI3K/AKT/mTOR) and AKT/ribosomal S6 kinase 2 (AKT/RSK2), lowering inflammatory responses. Furthermore, antioxidant defenses are enhanced through activation of the p62/nuclear factor erythroid 2-related factor 2 (p62/Nrf2) pathway, reducing oxidative stress and protecting joint tissues [71,72].

## 5. Future Research Directions: Artificial Intelligence (AI) and Bioactive Compounds from *A. annua* in COVID-19 Recovery

The integration of (AI) into natural product research is rapidly transforming the search for new drugs, especially regarding bioactive compounds from *A. annua* and their potential roles in COVID-19 recovery. AI-driven approaches now enable researchers to analyze vast datasets, predict compound-target interactions, and accelerate the identification of promising therapeutic agents, which is particularly valuable in the urgent context of emerging infectious diseases like COVID-19 [73]. Recent research shows that AI techniques, such as virtual screening and machine learning models, are being actively applied to compounds from *A. annua*. For instance, various studies [74,75] have utilized AI-driven molecular docking to evaluate phytochemicals in *A. annua*, specifically artemisinin derivatives and flavonoids, for their binding affinities against the SARS-CoV-2 main protease. This approach significantly accelerates the prioritization of drug candidates for laboratory testing. Additionally, advancements in machine learning in 2024, integrated with metabolomics and chemometrics, have enhanced the extraction and quality control of bioactive compounds from *A. annua*. This optimization allows for more efficient isolation of antiviral compounds with consistent potency [76]. These AI tools facilitate better formulation and standardization for therapeutic applications.

### 5.1. Recent Advances Have Demonstrated That AI Can

▪ Predict and rank bioactivity: Machine learning models can rapidly screen and rank the antiviral potential of *A. annua* compounds against SARS-CoV-2 molecular targets, such as the main protease (Mpro) and RNA-dependent RNA polymerase (RdRp) [77].
▪ Accelerate virtual screening: In silico (computer-aided) methods, including molecular docking and dynamics simulations, are increasingly used to evaluate how *A. annua*-derived flavonoids and terpenoids interact with viral proteins, significantly reducing the time and resources needed for experimental validation [78].
▪ Optimize extraction and formulation: AI tools help optimize extraction parameters for maximizing the yield and efficacy of bioactive compounds, ensuring that products are both potent and scalable for clinical use [73].

### 5.2. Recent Advances and Clinical Implications

- In Silico and in vitro studies: Multiple studies have used AI to identify lead compounds from *A. annua* with strong binding affinities to SARS-CoV-2 targets, supporting their further evaluation in laboratory and clinical settings [79].
- Clinical trials: Ongoing clinical trials are incorporating rapid AI-based assessment protocols to evaluate the efficacy of *A. annua* extracts and derivatives in COVID-19 patients, allowing for real-time data analysis and adaptive trial designs [80].
- Database integration: The establishment of large, AI-powered natural product databases is facilitating the sharing and analysis of global research findings, accelerating the translation of *A. annua* research into practical therapies [73].

### 5.3. Opportunities and Challenges Ahead

While AI has already begun to revolutionize the assessment and development of A. annua-based products for COVID-19 recovery, several challenges remain. These include the need for standardized data, improved interpretability of AI models, and the integration of multi-omics datasets to fully understand the mechanisms of action of complex herbal extracts. Nevertheless, the future is promising, with AI poised to further enhance the discovery, optimization, and clinical evaluation of bioactive compounds from *A. annua* and other medicinal plants [81].

## 6. Conclusions

*A. annua* and its bioactive compounds, particularly artemisinin and flavonoids, are promising therapeutic agents that support human health by combating oxidative stress-related complications such as inflammation and immune disorders. Additionally, artemisinin and its derivatives from *A. annua* are highly effective against microbial, malaria, and viral infections. AI-driven molecular docking and molecular dynamics simulations have identified artemisinin and its derivatives as promising candidates for fighting SARS-CoV-2 due to their strong and stable binding interactions with key SARS-CoV-2 proteins, including the main protease (Mpro) and spike protein. These compounds exhibit mechanisms that involve scavenging reactive oxygen species and modulating the immune response, both of which contribute to inhibiting viral replication and reducing inflammation. The synergistic effects of multiple phytochemicals in Artemisia enhance their broad-spectrum antiviral potential. Although initial in vitro and preclinical studies are promising, clinical evidence remains limited, highlighting the need for rigorous trials to determine effective dosages and long-term safety. Advances in extraction and formulation technologies are making Artemisia-based therapies more accessible, safe, and affordable. Moving forward, it will be critical to integrate clinical validation with ongoing biochemical and pharmacological research to fully realize Artemisia’s potential as a complementary or alternative treatment for COVID-19 and related viral infections.

## Figures and Tables

**Table 1:** Major bioactive molecules identified in *A. annua*.

Category	Compounds	Reference
**Sesquiterpene lactones**	Artemisinin, artemisinin I, II, III, IV, V, artemisinic acid, dihydro artemisinic acid, artemether, artemisinol, artannuin B, epoxy artennuic acid, voleneol, arteannoide B, C, H, I, L, M, P, U, W, X, Q, 3a-hydroxyartemisinic acid, 6,7-dehydroartemisinic acid,	[27,31,32,33]
**Phenolic acids**	Quinic acid, chlorogenic acid, caffeic acid, rosmarinic acid, ferulic acid, coumaric acid, 3-feruloquinic acid, 4-feruloquinic acid, 5-feruloquinic acid, 3,4-diferuloquinic acid, 3,5-diferuloquinic acid, 4,5-diferuloquinic acid, 3-caffeoylquinic acid, 4-caffeoylquinic acid, 3,4-di-caffeoylquinic acid, 3,5-di-caffeoylquinic acid, 3,5-di-O-caffeoylquinic acid, 4,5-di-O-caffeoylquinic acid, 3,5-caffeoyletherquinic acid, 4-caffeoyl-3,5-di-succinylquinic acid, diferulcaffeoylquinic acid,	[31,32,33,34]
**Flavonoids**	Artemetin, di-hydroartemisinin, rutin, luteolin, casticin, luteolin 7-O-glucosid, kaempferol, 8-methoxykaempferol, 3-methoxy-kaempferol glucoside, 3-O-glucoside of kaempferol, quercetin, quercetin 3-glucoside, 3-methyl-quercetin ether, isoquercetin, myricetin, myrcetin, vitexin, isovitexin, cirsilineol, chrysoeriol rutinoside, chrysosplentin, chrysosplenol D, eupatorine, rhamnetine, acacetin, chrysin, apigenin, apigenin 6-C-glucosyl-8-C-arabinoside, apigenin 6-C-arabinosyl-8-C-glucoside, astragalin, chrysosplenol C, cinaroside, isorhamnetin, syringetin, kirsiliol, kirsimaritin, tamarixetine, quercimeritin, jaceidin, retina, laricitrin, micanine, marnsetin, patulentin glucoside, chrysosplenetin.	[31,32,33,34,35]
**Coumarins**	Scopoletin, coumarin, cis-melilotoside, scopoline, trans-melilotoside, esculetin, tomentin, isofraxidine.	[31,32]
**Terpenes**	1,8-Cineole, germacrene-D, α-pinene, camphene, borneol, camphor, carvone, limonene, α-terpinene, myrtenol, caryophyllene, sabinene, linalool, eugenol, β-myrcene, β-thujone, 4-terpineol, piperitone, α-longipinene, α-copaene, γ-muurolene, β-pinene, β-selinene, bicyclogermacrene.	[28,29]

## Data Availability

Data are contained within the article.
